# Supplementary material for: How have urban housing preferences developed in response to the COVID-19 pandemic? A case study of Vienna
Source: PLoS One. 2025 May 30;20(5):e0322629. doi: 10.1371/journal.pone.0322629 (PMC12124504; doi:10.1371/journal.pone.0322629)
Supplement: Attachment — Submitted filename: Braesemann et al (2025) Housing Preferences RESPONSE LETTER.pdf [file pone.0322629.s001.pdf]

How have urban housing preferences developed in response to the Covid-19 pandemic? A case study of Vienna

by Fabian Braesemann, Jan Kluge, and Hanno Lorenz

PLOS ONE
(Manuscript Ref. No.: PONE-D-24-30670)

Revision: reviewer response letter

In the following, please find our point-by-point responses to the comments raised by the reviewers. In the revised manuscript we mark new text with a reference to the corresponding reviewer comment to facilitate a straight-forward review of our changes.

With best regards,

Fabian Braesemann
— Corresponding author —

Editor comments

E1: Overall evaluation #1

Dear Dr. Braesemann,

Thank you for submitting your manuscript to PLOS ONE. After careful consideration, we feel that it has merit but does not fully meet PLOS ONE's publication criteria as it currently stands. Therefore, we invite you to submit a revised version of the manuscript that addresses the points raised during the review process.

In particular, I encourage the authors to address the issues raised by Reviewer 2 regarding the methodologies and potential confounding factors for the analysis.

Our response:
Dear Prof. Maurizio Fiaschetti,

Thank you for the opportunity to revise the paper. We have engaged thoroughly with the reviewers' comments and revised the manuscript accordingly. We believe we have addressed every point that has been raised.

Most importantly, we provided additional details on the data collection and preparation as it was requested by both Reviewer 1 and Reviewer 2. Thanks to the helpful comments from Reviewer 2 with respect to the econometric methodology, we could improve the main regression model by

using a log-transformed dependent variable and by using a regression model with clustered standard errors. Both these steps have resulted in a more accurate model with higher R^2 values. This also had an effect on the second comment by Reviewer 2 as the public transport coefficient estimate is, in fact, not statistically significant, as we could show thanks to the improved methodology.

In addition, we have provided additional contextualisation on other factors that could possibly have had an impact on the rent premium in Vienna during the observation period.

Overall, we feel that our manuscript has been improved thanks to the helpful comments made by the reviewers, and we are grateful for the time the have taken to review the manuscript.

Please find our detailed response to each comment below. The reviewers' comments are in black. Our response is below them in blue.

Please note that we have added a coding scheme to all new text in the manuscript. For example, **(R1.2)** refers to new text that addresses Reviewer 1's second comment. This way, it should be straightforward for the reviewers to see how and where we have incorporated their feedback.

Best regards,

Fabian Braesemann (on behalf of all authors)

Reviewer 1

R1: Overall evaluation

The research article titled "How have urban housing preferences developed in response to the Covid-19 pandemic?" aims at exploring how the Covid-19 pandemic have influenced housing preferences in Vienna. By analysing a large database of apartment listings from an online real estate platform, the authors identify changes in rent premiums for various amenities and features, highlighting a shift towards preferences in the analysed period.

The study addresses a highly relevant topic, that fits in the current literature about the impacts of the Covid-19 pandemic on urban living and working conditions. The literature review on the relationship between the changes induced by the pandemic and the housing prices is well written and complete. The analysis is rigorous, and the figures are clear. Moreover, the authors clearly state the limitations of their analysis, highlighting the possible bias in the data collection, as well as the lack of causal claims of the results.

Our response:

Thank you for your evaluation and for highlighting the relevance of the topic.

Please find below our response to the suggestions for improvements made, detailing how we incorporate your feedback in the manuscript, which we think has benefited from it.

Major comments

R1.1: Data

The data source, such as the procedure followed to obtain the analysed data is not fully clear. The authors should deeply describe the text-mining procedure followed to get the data, as well as the data cleaning procedure. Without a clear description of the procedures, it is difficult to assess the reliability of the data.

Our response:

Thanks for pointing out the need to provide more details on the data collection and preparation. In the revised version of the manuscript, we now provide more details on each step, including naming the platform we used to collect the data (willhaben.at), a detailed description on the workings of the web scraper, the variables we extracted, and the steps undertaken in cleaning the data before analysis (see also our response to R2.1).

R1.2: Results

It is necessary deep the reasons why in the period 2021/2022 the availability of transport network leads to a reduction of the rent (significant and negative effect). This result is unexpected and should be carefully interpreted, otherwise the entire analysis loses credibility.

Our response:

Thank you for the critical comments. This result is indeed surprising as proximity to public transport is usually one of the main drivers of rents. Thanks to the improved econometric methodology (see our responses to R2.3 and R2.4), this effect largely disappeared. The public transport coefficient estimate is not statistically significant anymore. Accordingly, we have removed our previous discussions of this finding.

Minor comments

R1.M1:

Even if the authors deeply describe the results with extensively description of Vienna's housing dynamics and specific characteristics, the results seem quite city-specific. The focus on a specific case study (together with the lack of a causal claim) limits the external validity of the study. For this reason, I would suggest to partially change the title of the research mentioning the name of the city.

Our response:

We have amended the study's title and included a sub-title "A case study of Vienna".

R1.M2:

At page 17 the authors write that “we have used information from the previous analyses to guide the inclusion of variables”. It is necessary to cite the articles (at least some of them).

Our response:
This must have been a misunderstanding. We did not mean to imply that we used information or data from any external previous studies. Instead, what we meant to say was that we used the information from the analyses presented in the results section of this manuscript to inform the feature selection of the regression model. Informing feature selection of the regression model is also the motivation for the use of more exploratory non-parametric machine learning models such as the LASSO model (see our response to R2.3).
We have amended the wording in the results section to avoid any misunderstanding.

Reviewer 2

Major comments

R2.1: Data Clarity and Representativeness

The data presented in the paper is insufficiently described. There is too little information about the company providing the data, and no discussion regarding its representativeness. Additionally, there is a lack of detail about how the data is structured and how it was obtained, aside from a brief mention that it was scraped and abstracted using text recognition. Crucial details, such as the precise time periods during which the data was collected, are also missing. The paper mentions that data was collected over six months in 2018 and during fall/winter 2021/2022, but it is unclear whether this also covers a six-month span. This lack of clarity on the temporal aspect of the data collection raises concerns, as differences in the time periods may explain observed variations, potentially independent of the pandemic's effects, such as seasonal effects.

Our response:
We thank the reviewer for pointing out these potential issues. We have made an effort to describe the data collection in more detail (see also our response to **R1.2**). In the updated version of the manuscript, we mention the platform from which we collected the data (willhaben.at), and we state the exact periods of data collection. These do not overlap completely: the data collection in 2018 was conducted between the second and fourth quarter of the year, while the data collection in 2021/22 spanned Q4 2021 to Q2 2022. However, we do not believe that seasonal differences will have had a substantial influence on the findings in contrast to the main change between the two observation periods brought about by the pandemic. Still, we have added this as a potential limitation to the limitations section 5.4.

R2.2: Contextual Factors Beyond the Pandemic

The paper does not sufficiently discuss other significant events that occurred during the studied period, aside from the pandemic, which could have affected the results. For instance, the Russian invasion of Ukraine led to sanctions that impacted gas prices. This may explain the increase in premiums for floor heating, particularly in regions like Austria, where, to my knowledge, Russian gas imports continued. A discussion of these additional factors is necessary to avoid attributing all observed changes solely to the pandemic.

Our response:
Thank you for your suggestion. We now discuss several aspects that have possibly affected the rental market and which need to be discussed accordingly. Next to the war in Ukraine, we now discuss the location of new dwellings, construction costs, and regulatory and weather conditions as the impact of the new home-office rules on our findings. Some of these factors could explain the heterogeneity between districts – such as the location of new dwellings. We have added paragraphs related to these factors at several points in the manuscript, including a new subsection in the Discussion.

R2.3: Statistical Models and Methodology

The statistical models, particularly the LASSO model and conventional regression models, are poorly described. A more detailed and intuitive discussion of the properties of these models is required. Furthermore, the paper lacks essential information regarding model implementation, such as whether standard errors were clustered, which is a critical omission.

Our response:
We are particularly grateful to the reviewer for pointing out the possible need to use a log transformation (see our response below) and cluster standard errors in the regression model.
We compared the simple OLS models we ran in the data analysis for the original version of the manuscript (only district-level fixed effects) with models that include both district-level fixed effects and cluster standard errors on the district level.
Indeed, we find that our original model specification has possibly underestimated standard errors. We updated the regression table in Figure 3C completely. The updated model in Figure 3C of the manuscript should be more accurate. While most coefficient estimates are unaffected by the changed model specification, the slightly unexpected strong effect public transport seemed to have has mostly disappeared.
Thanks again to the reviewer for pointing out to us that we should use a model that accounts for clustered standard errors.

We have also added a more intuitive explanation of the LASSO model and why we have included it in the manuscript.

R2.4: Lack of Descriptive Statistics

The absence of descriptive statistics for the data severely limits the ability to assess the models' reasonableness. For example, there is no information on the distributional properties of key variables, such as rent, which makes it impossible to determine whether a level or log transformation would be more appropriate for the dependent variable. Similarly, without details about the explanatory variables, it is difficult to evaluate the relevance and reliability of the results. Providing descriptive statistics and an appropriate discussion of the data's properties is essential for a more thorough assessment of the models and findings.

Our response:
The reviewer makes an important point here. We have created a new figure in the appendix showing the distribution of the dependent variables used in the regression models: monthly rent in EUR and monthly rent in EUR per square metre with and without a logarithmic scale. The figure shows that monthly rent in EUR is indeed log-normally distributed. Accordingly, we have updated the regression models (1) and (3).
